# Understanding the Impact of COVID-19 on Angioplasty Service and Outcome of Patients Treated for Chronic Limb-Threatening Ischaemia: A Single-Centre Retrospective Cohort Study

**DOI:** 10.3390/biomedicines11072034

**Published:** 2023-07-19

**Authors:** Alexander D. Rodway, Jenny Harris, Lydia Hanna, Charlotte Allan, Felipe Pazos Casal, Ciara Giltinan, Ali Dehghan-Nayeri, Andre Santos, Martin B. Whyte, Nikolaos Ntagiantas, Ivan Walton, Richard Brown, Simon S. Skene, Ajay Pankhania, Benjamin C. T. Field, Gary D. Maytham, Christian Heiss

**Affiliations:** 1 Department of Vascular Medicine, Surrey and Sussex Healthcare NHS Trust, Redhill RH1 5RH, UK; 2 Department of Vascular Surgery, University Hospital Sussex NHS Trust, Brighton BN2 5BE, UK; 3 Department of Adult Nursing, School of Health Sciences, Faculty of Health and Medical Sciences, University of Surrey, Guildford GU2 7XH, UK; 4 Department of Vascular Surgery, Imperial College Healthcare NHS Trust, London SW7 2BX, UK; 5 Department of Clinical and Experimental Medicine, School of Biosciences, Faculty of Health and Medical Sciences, University of Surrey, Guildford GU2 7XH, UK; 6 St. George’s Vascular Institute, St. George’s University Hospitals NHS Foundation Trust, London SW17 0QT, UK

**Keywords:** day-case angioplasty, critical limb ischaemia, peripheral artery disease

## Abstract

We evaluated the impact of COVID-19 restriction on the angioplasty service and outcome of chronic limb-threatening ischaemia (CLTI) patients undergoing lower-limb angioplasty in a UK secondary care setting. Consecutive patients were analysed retrospectively. Pre-COVID-19 (08/2018–02/2020), 106 CLTI patients (91% Fontaine 4; 60% diabetes mellitus) and during COVID-19 (03/2020–07/2021) 94 patients were treated (86% Fontaine 4; 66% diabetes mellitus). While the average monthly number of patients treated did not change, the proportion of day cases significantly increased (53% to 80%), and hospitalised patients decreased. Patients treated in ≤14/5 days after referral significantly increased to 64/63%. Kaplan–Meier survival analysis (30-day/1-year) showed that neither wound healing nor mortality were significantly changed during COVID-19. In day cases, 1-year but not 30-day major amputations significantly increased, and clinically driven target-lesion revascularisation decreased during COVID-19. One-year mortality was significantly worse in hospitalised compared to day cases (14% vs. 43%) at similar wound healing rates (83% vs. 84%). The most frequent known cause of death was infectious disease (64%), while cardiovascular (21%) was less frequent. Despite COVID-19 restrictions, a safe and effective angioplasty service was maintained while shortening waiting times. Very high mortality rates in hospitalised patients may indicate that CLTI patients need to be referred and treated more aggressively earlier.

## 1. Introduction

Peripheral artery disease (PAD) is a growing medical problem worldwide [1], affecting as many as one in four adults in developed countries [2]. The prevalence increases with age, and in diabetes it may be present in up to 50% of patients [3]. In its most severe form, chronic limb-threatening ischaemia (CLTI), the level of blood flow reaching the limb is inadequate for baseline metabolism, with patients suffering rest pain and restricting wound healing, often leading to tissue loss and limb amputation. A rapid response to a diagnosis of CLTI is crucial to prevent further tissue loss and save limbs [4].

Rates of 1-year all-cause mortality in patients hospitalised with CLTI are alarmingly high (16–35%) [5]. This is associated with a 1-year risk of amputation of between 5 and 57% [5]. A recent meta-analysis with a mean follow-up of 6.3 years showed that 21% of symptomatic PAD patients with claudication progress to CLTI, with 4–27% undergoing amputations [6]. In nearly half of patients with diabetic foot disease, PAD is present as diagnosed by low ankle brachial pressure index (ABPI) [7]. Supporting the class 1 indications for revascularisation in CLTI [8,9], a recent meta-analysis showed that even in patients with diabetes, revascularisation is effective to promote wound healing and prevent amputation in CLTI [10].

In March 2020, the rapidly evolving situation regarding the COVID-19 virus and lockdown led to rapid and significant changes in hospital services. The guidance issued by the president of the Vascular Society (along with GIRFT and the Specialist Commissioners) stated ‘Where possible, only urgent outpatients should be seen, and virtual clinics should be considered. Elective arterial surgery and venous surgery should be deferred. Those legs immediately threatened require urgent intervention. Others may be diverted to a hot foot clinic for further assessment. Interventional radiological approaches may allow more appropriate utilisation of scarce high dependency beds. There may be situations where primary amputation may be more appropriate than complex revascularisations, multiple debridements and potential prolonged hospital stay’.

Here, we evaluated the impact of COVID-19 service restrictions on our angioplasty service and the outcome of CLTI patients undergoing lower-limb angioplasty at East Surrey Hospital.

## 2. Materials and Methods

### 2.1. Study Design, Setting and Patients

We performed a single-centre retrospective analysis of consecutive patients undergoing lower-limb endovascular revascularisation from July 2018 to February 2020 (‘pre-COVID-19′) and March 2020 to July 2021 (‘COVID-19′) at East Surrey Hospital (Redhill, UK). The hospital is a secondary-care district general hospital serving a local population of 744,000 people supported by the vascular surgery hubs at Royal Sussex County Hospital (Brighton and Sussex Medical School, Brighton, UK) and St. George’s University Hospital (London, UK). The team consists of vascular surgeons and interventional radiologists on joint appointments and angiology and diabetology clinical academics on joint appointments with the University of Surrey Medical School (Guildford, UK). We compared patient and procedural characteristics, technical success, peri-procedural complications, 30-day and 1-year wound healing, mortality, major amputations and clinically driven target-lesion revascularisations (TLR) of angioplasties performed in outpatients as day cases and hospitalised patients.

Our vascular spoke centre specialises in day-case-based revascularisations in CLTI patients as part of a multidisciplinary foot salvage service [11]. Independent of COVID-19, we aimed to follow the PAD quality improvement framework (QIF) guidance for ‘non-admitted’ and ‘admitted’ pathways for CLTI, aiming at revascularisation in less than 14 days or less than 5 days, respectively. Of note, none of the hospitalised patients in the current study were primarily admitted through the vascular service. Patients presented to the vascular team with leading severe chronic ischaemia and deemed to require revascularisation in less than 5 days were discussed with vascular hub and urgently transferred if faster revascularisation could be achieved by doing this. Patients with acute limb ischaemia were also immediately transferred to a vascular hub.

### 2.2. Patient Assessment and Procedure Planning

All patients were presented and discussed at our multi-disciplinary team (vascular surgery, angiology and interventional radiology in attendance) or multi-disciplinary diabetic foot team (vascular surgery, angiology, diabetology, microbiology, podiatry and tissue viability in attendance) meetings. The diagnosis of PAD was based on ABPI < 0.9 with concordant Doppler waveforms [12] and the classification into clinical stages made according to the Fontaine Classification (Fontaine II for claudication, Fontaine III for rest pain or Fontaine IV when rest pain or ischaemic ulcers/gangrene were present and plausibly related to ischaemia. CLTI was defined as Fontaine III and IV. In case of incompressible ABPI (>1.2), discordance with the Doppler waveform or borderline values that appeared implausible compared to the clinical picture, toe-brachial index (TBI) or intra-arterial angiography were performed [8,13,14]. Indications for revascularisations were based on current PAD treatment guidelines [8,13,14], including short-distance lifestyle-limiting claudication not responding to or amenable to exercise therapy, rest pain, non-healing ulcer or gangrene. Individualised optimal revascularisation strategy (angioplasty vs. open surgery), surgical risk, technical feasibility and procedure planning were assessed by the team’s interventionalists and vascular surgeons based on clinical characteristics, symptoms and imaging including Duplex ultrasound and CT angiograms [14]. Finally, the multi-disciplinary treatment plan, risks and expected benefits were discussed with the patient, informed consent signed and procedure scheduled.

### 2.3. Day-Case Criteria and Procedure Preparation

Outpatients were accepted for ‘day case’-based endovascular intervention if all the following criteria were met: body mass index < 35 kg/m^2^, American Society of Anesthesiology score < IV, eGFR > 29 mL/min, sheath < 7F needed for procedure, not socially isolated with <1 h drive to hospital, telephone available with responsible adult present overnight and no anti-coagulation requiring bridging. In individual outpatient cases, patients with lower eGFR or higher ASA were accepted or procedures with 7F performed with special arrangements. When patients fulfilled all medical criteria but were not able to arrange for a responsible adult to stay with them overnight or requested to stay in, we admitted the patients ‘overnight’. If the pre- or post-procedural risk was deemed high by the interventionalists or open revascularisation required, then patients were referred to be treated at one of the partner vascular surgery hubs.

### 2.4. Peri- and Post-Procedural Management

Metformin was paused 24 h prior to procedure. Warfarin was paused to reach a target INR < 1.5. Direct-acting oral anticoagulants were paused for 48 h prior to the procedure and restarted on the next day. Aspirin and clopidogrel were not paused. Strict fasting status was not required, and patients were allowed a small breakfast up to 3 h before the procedure.

All procedures were performed by an experienced consultant angiologist or interventional radiologists according to current treatment guidelines aiming at establishing at least single-vessel straight-line flow to the foot. In cases of crural artery disease, preference was given to the artery supplying the angiosome in which the ulcer or gangrene was present. All patients received a Duplex ultrasound scan of the access sites on the table by the interventionalist. Access site punctures were mostly performed under ultrasound guidance. Technical success was defined as the visually successful treatment of the target lesion(s) with less than 30% remaining stenosis. If possible, vascular occlusion devices were used (Angioseal, St Jude Medical, MN, USA). A target procedure finishing time was set at 14:00 to allow sufficient observation time in the day-case unit prior to discharge from hospital or to the ward.

Post-procedure, patients lay flat for 1 h followed by 1 h of bed rest with elevated head, followed by a light snack and drink if desired and discharge after a groin check at 4 h. If access was difficult or there was superficial bleeding visible through the transparent dressing, a Duplex ultrasound was performed to exclude a haematoma or pseudo-aneurysm. Unless there was a contra-indication, patients were started on dual antiplatelet therapy consisting of aspirin 75 mg and clopidogrel 75 mg for 1–3 months (3 months only when drug eluting stents were implanted) with a clopidogrel loading dose of 300 mg. In case of oral anti-coagulation, we added only clopidogrel temporarily. In addition, medications were reviewed and adapted towards optimal medical management if required.

All patients were scheduled within a month of discharge for a Duplex ultrasound examination of the access site and intervened vessel segment together with ABPI and vascular clinic review. Wound care continued throughout within our network or local podiatry.

### 2.5. Clinical Outcome Assessment

We recorded wound closure, mortality, major amputation or clinically driven target-lesion revascularisation (TLR) within 30 days and 1 year following angioplasty based on electronic patient records at East Surrey Hospital. When patients died during the time period, the electronic patient records were searched for the medical examiner’s note and the cause of death recorded.

### 2.6. Statistical Analyses

Data are presented as mean and standard deviation. Mean values were compared using one-way ANOVA, and if *p* < 0.05, consecutive Bonferroni post hoc between-group comparisons were considered. Data for which normality could not reasonable assumed and data and categorical parameters were compared using the Mann–Whitney U test. Wound closure, mortality, major amputation or TLR within 30 days and 1 year following angioplasty were assessed with Kaplan–Meier survival analysis and the log-rank (Mantel–Cox) test. Analyses were performed with SPSS 28 (IBM, Portsmouth, UK).

## 3. Results

### 3.1. Baseline and Procedural Characteristics

**Figure 1:**
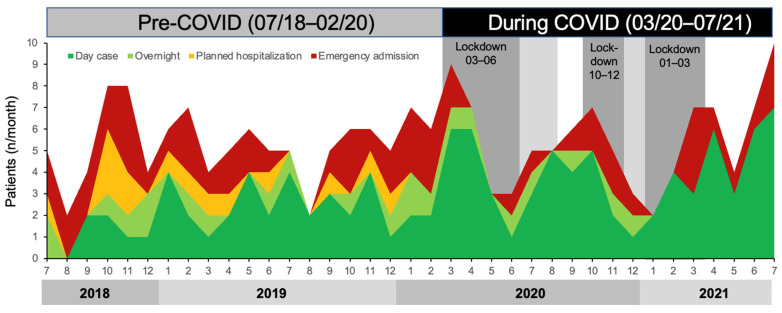
Evolution of numbers of patients who underwent endovascular revascularisations at Surrey and Sussex Healthcare NHS Trust 07/2018–07/2021.

One hundred and six patients with CLTI were treated pre-COVID-19 (91% Fontaine 4; 60% diabetes mellitus), and ninety-four (86% Fontaine 4; 66% diabetes mellitus) were treated during COVID-19. See Figure 1 for time-course of patient numbers. Overall, the clinical characteristics did not differ except for a statistically significant higher percentage of patients using statins during COVID-19 (Table 1). With regards to procedural characteristics (Table 2) during COVID-19, the mean treated lesion length was larger (pre: 141 ± 97 mm vs. during: 253 ± 158 mm, *p* < 0.001), and stented segments shorter (pre: 135 ± 59 mm vs. during: 87 ± 82 mm, *p* = 0.005). Post-procedural ultrasound surveillance scans were performed in 82% pre-COVID-19 and 84% during COVID-19. The post-procedural ABPI was significantly higher (pre: 0.81 ± 19 vs. during: 0.87 ± 0.20, *p* = 0.045).

### 3.2. COVID-19-Related Changes in Angioplasty Service

While the average monthly number of patients treated did not change (pre-COVID-19: 5.3 ± 1.7, COVID-19: 5.0 ± 2.1, *p* = 0.612), the day-case-based outpatient procedures increased (53% to 80%, *p* < 0.001). Within these, the number of same-day discharges (39% to 72%, *p* = 0.009) increased. Conversely, the overall treatment of hospitalised patients more than halved (47% to 20%, (*p* < 0.001). The numbers of both planned urgent hospitalisations (13% to 0%, *p* = 0.010) and emergency admissions (34% to 20%, *p* = 0.010) were lower during COVID-19 compared to pre-COVID-19. Most hospitalised patients were admitted via the emergency department with foot pain, infected foot or leg wounds. Planned urgent hospitalisations were initiated in outpatients by the vascular team for combined urgent wound debridements or minor amputations of necrotic toes and combined with revascularisation.

The mean interval between multi-disciplinary treatment decision and angioplasty procedure significantly decreased during COVID-19. The percentage of outpatients treated within 14 days after referral, as suggested by the PAD QIF, increased from 39% (average waiting time: 25 ± 20 days) to 65% (15 ± 13 days, *p* = 0.009) and hospitalised patients treated within 5 days from 44% (11 ± 13 days) to 63% (5 ± 6 days, *p* = 0.008). The average length of stay also significantly decreased by a third during COVID-19 (22 ± 20 days vs. 7 ± 19 days, *p* < 0.001).

### 3.3. Effect of COVID-19 on 30-Day and 1-Year Outcomes

As summarised in Table 3, there were no significant differences in outcomes pre-COVID-19 and during COVID-19 at 30 days in terms of wound healing, mortality, major amputation or TLR. In the pre-COVID-19 period, the 30-day wound healing was significantly greater in day cases, compared to hospitalised patients, but 30-day mortality, major amputation and TLR rate were similar between groups.

**Figure 2:**
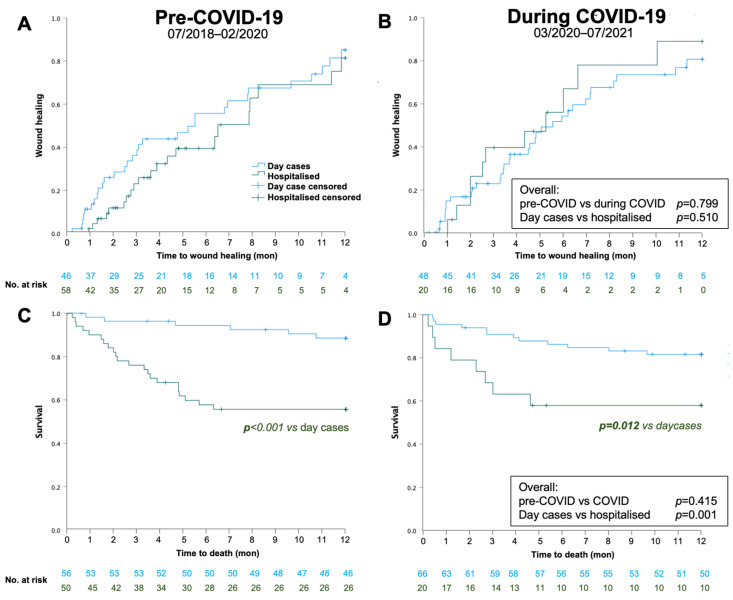
Kaplan–Maier survival analysis for 1-year wound healing (**A**,**B**) and survival (**C**,**D**) pre-COVID-19 (**A**,**C**) and during COVID-19 (**B**,**D**).

**Figure 3:**
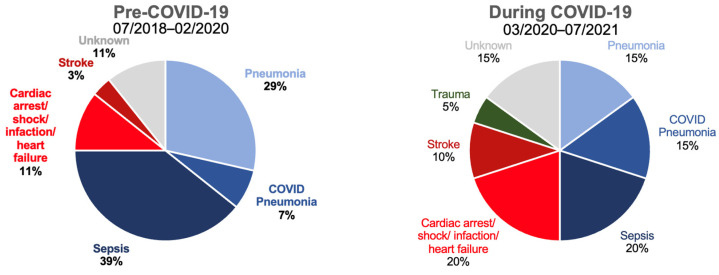
Cause of death pre- and during COVID-19 periods (*n* = 28 and *n* = 20) as noted on medical examiner’s report.

There were no statistically significant differences between 1-year wound healing or mortality between the pre-COVID-19 and COVID-19 periods. However, mortality was strikingly higher in hospitalised patients compared to outpatient day cases independent of COVID-19 (43% vs. 14%; Table 4, Figure 2). A total of 48 patients died (pre-COVID-19: *n* = 28, during COVID-19: *n* = 20). Figure 3 shows the distribution according to the documented cause of death. Surprisingly, the majority of known causes of death were infectious disease, namely sepsis, pneumonia and COVID pneumonia, accounting for 75% of all deaths pre-COVID-19 (*n* = 21) and 50% during COVID-19 (*n* = 10). These were more frequent than cardiac or stroke which together only accounted for 14% (*n* = 4) and 30% of deaths (*n* = 6, *p* = 0.831, pre vs. during COVID-19).

**Figure 4:**
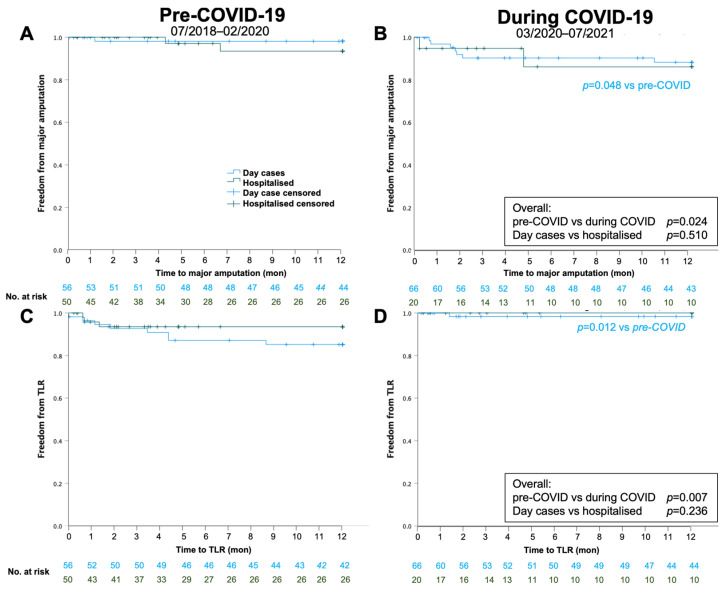
Kaplan–Maier survival analysis for 1-year major amputation (**A**,**B**) and clinically driven target-lesion revascularisation (TLR; **C**,**D**) pre-COVID-19 (**A**,**C**) and during COVID-19 (**B**,**D**).

During COVID-19, major amputations were significantly more frequent and TLR significantly less frequent, compared to pre-COVID-19. This was only statistically significant in day-case patients (Table 4, Figure 4).

We also performed a Cox regression analysis to determine major contributors for mortality (Table 5). This analysis showed that increasing age, being hospitalised and a diagnosis of coronary artery disease contributed to mortality. However, none of male sex, being treated during COVID-19, baseline ABPI, time to procedure, diabetes mellitus or statin use were associated with mortality.

## 4. Discussion

The current data show how COVID-19 restrictions affected endovascular treatments and outcomes of CLTI patients in an NHS district general hospital. Care pathways were changed to protect patients from COVID-19 infection and to open bed capacity for COVID-19 patients. This negatively impacted resources available for non-COVID-19 patients with staff redeployment outside usual specialist roles. However, the Vascular Society recognised that ‘CLTI/diabetic foot … Those legs immediately threatened require urgent intervention’ and that ‘Interventional radiological approaches may allow more appropriate utilisation of scarce high-dependency beds’. Following this guidance and contrary to frequent reports in news media, in our service, the number of CLTI patients treated remained stable during COVID-19, and waiting times significantly improved towards nationally recommended targets that were set before COVID-19 but were upheld during COVID-19 (revascularisation ≤ 14 days via non-admitted pathway and ≤5 days via admitted pathway for CLTI).

We show that the number and proportion of day cases increased, while angioplasties in hospitalised patients decreased. This occurred in part because patients who would usually have been treated as inpatients, particularly through urgently planned hospitalisation, were instead managed as day cases. Day-case procedures remained possible as they followed a green pathway with mandatory COVID testing in all patients separated from the rest of the hospital and because some staff performing MDTs and interventions were not re-deployed and were able to continue their vascular duties. As depicted in Figure 1, even during COVID-19 lockdowns, individual day-case patients were admitted overnight on a ‘COVID free’ ward when there was no other adult living with them, and lockdown prohibited anyone staying with them. In all cases, the clinical severity of CLTI was so high that the patients accepted the increased risk of contracting COVID-19 despite precautions. Fortunately, our data now indicate that day-case patients (same-day discharge and overnight) were not at increased peri-procedure risk when treated during the COVID-19 period as indicated by the similar 30-day outcomes pre-COVID-19 compared to during COVID-19 (Table 3). There were two and three COVID pneumonia deaths pre- and during COVID-19 periods, respectively. However, only one patient died during first lockdown with COVID-19 pneumonia at 14 days after the day-case procedure which would be compatible with having contracted COVID-19 infection in the hospital. The remaining deaths occurred in >84 days post-procedure and are unlikely linked to the hospital stay. Our similar 1-year mortality rates pre- and during COVID-19 periods provide further reassurance. In addition, the 1-year amputation-free survival of outpatients was 21% which is similar to the best endovascular treatment first arm of the BASIL-2 trial [15], and major amputation was 7%, which is similar to the BEST-CLI trial [16].

Day-case-based angioplasty is becoming increasingly common, and we have previously reported our experience and 30-day outcomes [11]. Potential risk factors for outpatient procedures in CLTI were recently discussed [17]. Our current data support the safety and efficacy of day-case angioplasties [11] even in patients with CLTI [17], as the complication rates were low and technical and clinical success high, with increased ABPI in most patients and high rates of wound healing independent of the COVID-19 period. The complications, 30-day mortality, major amputation and TLR did not differ between day cases and hospitalised patients. In addition, day cases had higher wound healing rates already at 30 days compared to those who were hospitalised and despite longer waiting times. Of note, all patients were followed up, including review and optimisation of medical therapy, which likely contributed to the outcomes [14] highlighting the importance of multidisciplinary case discussions among vascular specialist [18] and the need for day-case procedure being accompanied by a follow-up program including optimal medical management [14].

The most striking finding of the current analysis was the extremely large 1-year mortality (43%) in patients who were hospitalised, independent of the COVID-19 period. This aligns with published data in German hospitals [5]. Our data suggest that the need for hospitalisation identifies CLTI patients at very high risk for mortality. We believe that this indicates that CLTI patients need to be identified and treated early to avoid urgent hospitalisation as supported by primary-care data showing that early detection of ulcers and prompt referral improve amputation-free survival [19]. Note that ulcers were present for 138/187 days. It is noteworthy that cardiovascular and cerebrovascular causes of death were less common in our cohort than inflammatory disease outside the leg, presenting as sepsis and/or pneumonia. A recent meta-analysis has also shown lower-than-expected cardiovascular-related mortality [6], reporting a 5-year cumulative incidence of mortality of 27% in symptomatic PAD patients but only 13% cardiovascular mortality, supporting our findings. While in some of the cases, the infected foot wound might have been the focus of sepsis, our finding leads to the question of whether, and how, CLTI could be linked with increased systemic susceptibility to inflammatory disease. Possible explanations might include a link between CLTI and frailty, and/or increased nosocomial exposure to respiratory pathogens, and/or frequent antibiotic treatment of infected wounds altering bacterial flora and selecting for pathogenic and/or resistant strains.

### Limitations

This is a single-centre, retrospective, observational study, the data for which reflect unselected consecutive cases of CLTI requiring angioplasty. Our findings may thus not be representative of other institutions, as our multidisciplinary team, unusually for the UK, includes a clinical academic interventional angiologist who was not re-deployed during COVID-19. The latter likely differs from what other centres experienced as it allowed us to continue a functioning angioplasty service despite COVID-19 restrictions. Another limitation relates to the fact that, notwithstanding the overall similarities of baseline characteristics, our data cannot show if the increase in proportion of day-case procedures was fully explained by altered clinical decision making during COVID-19 pandemic restrictions or by subtly altered case-mix. The observation that lesion length was significantly longer during COVID-19 compared to pre-COVID-19 supports that there was a change in case-mix. This might include a smaller proportion of presentations being to the Accident and Emergency department or desire to avoid admission, with more people presenting instead via vascular/foot clinic and treated as outpatients. A further possibility is that there was an increase in frequency of patients being referred directly to regional vascular hubs for emergency amputations, which would have escaped our analysis. However, our observation of lower numbers of hospitalised emergency admissions for acute feet aligns with international trends of lower admissions for foot ulcers during COVID-19 [20,21,22]. A French nationwide study reported lower hospitalisation rates in diabetic foot ulcer patients along with the lower revascularisation rates [21], which aligns with our current data and may point towards overall lower access to health care during the COVID-19 lockdowns. Different to the French study, we observed unchanged revascularisation rates but higher major amputation rates, particularly in day cases. We speculate that, during lockdown, patients with higher amputation risk that would have otherwise been admitted were treated as day cases or might have presented too late for foot/leg salvage attempts by revascularisation. An alternative explanation is that, to reduce cumulative risk of SARS-CoV-2 exposure during frequent hospital visits for dressings, investigations and repeat angioplasties with relatively lower chances of success, clinicians might have had a lower threshold for recommending amputation during the COVID-19 lockdowns as also recommended by the Vascular Society. Our data suggest that patients were not at increased procedure-related risk when treated during COVID-19, as indicated by the similar 30-day outcomes (Table 3). Our similar 1-year mortality rates before and during COVID-19 provide further reassurance. A further limitation of the study is that the wound size was not documented. Differences in mortality between hospitalised and outpatients may be due to more severe wounds. Finally, the time from referral to revascularisation in hospitalised patients in the current study does not necessarily reflect a typical population of ‘admitted’ pathway CLTI patients. As our hospital is a vascular spoke, patients with acute and rapidly progressing ischaemia, and therefore requiring urgent revascularisation for this reason within 5 days, are typically discussed with and transferred to the vascular hub hospital for emergency treatment. One could argue that the required time to revascularisation of our patients should be rather evaluated with the less-than-14-days target like the ‘day cases’.

## 5. Conclusions

In conclusion, our data show that, while adapting to COVID-19 restrictions, we maintained a safe and effective angioplasty service and shortened our waiting times. Very high mortality rates in patients after hospitalisation, unrelated to the COVID-19 period, indicate that CLTI needs to be detected and referred to vascular specialists earlier to be treated pro-actively and avoid disease exacerbation requiring hospitalisation. Further development of day-case-based angioplasties as part of integrated foot salvage services may be the way forward to meet this growing medical need.

## Figures and Tables

**Table 1:** Clinical and demographic characteristics of patient population pre- and during COVID. Values are mean and standard deviation (or percent of total if indicated); *p* values are from *t*-test or Mann–Whitney U test.

	Pre-COVID-19	During COVID-19	*p*
	07/2018–02/2020	03/2020–07/2021	
n (*n*)	106	94	
Sex (m/f)	61/45	66/28	
Day case-based outpatients	56	75	<0.001
- Same day discharge (*n*)	41	68	0.009
- Overnight (*n*)	15	7	0.009
Hospitalised (*n*)	50	19	<0.001
- Planned urgent hospitalisation (*n*)	14	0	0.010
- Emergency admission (*n*)	36	19	0.010
Time to procedure (d)	19 ± 18	15 ± 13	0.129
- daycases (d)	25 ± 20	18 ± 13	0.009
- daycases ≤ 14 days	39%	64%	0.291
- hospitalised (d)	11 ± 13	5 ± 6	0.018
- hospitalised ≤ 5 days	44%	63%	0.291
Age (years)	75 ± 12	74 ± 10	0.310
Haemoglobin (mg/dL)	119 ± 20	124 ± 20	0.060
Leucocytes (/dL)	9.5 ± 4.0	10.3 ± 10.2	0.475
Platelets (/dL)	323 ± 127	288 ± 116	0.052
Estimated glomerular filtration rate (mL/min)	55 ± 19	64 ± 23	0.004
Total cholesterol (mg/dl)	4.1 ± 1.1	4.4 ± 1.2	0.196
Low-density lipoprotein cholesterol (mg/dL)	2.0 ± 0.9	2.2 ± 1.0	0.195
High-density lipoprotein cholesterol (mg/dL)	1.4 ± 0.4	1.3 ± 0.4	0.194
Triglycerides (mg/dL)	1.5 ± 0.8	1.8 ± 0.9	0.031
International Normalized Ratio	1.2 ± 0.4	1.2 ± 0.4	0.342
HbA1c (mmol/mol)	43 ± 8	56 ± 23	0.072
Systolic blood pressure (mmHg)	147 ± 22	151 ± 21	0.242
Diastolic blood pressure (mmHg)	82 ± 13	83 ± 11	0.637
Fontaine III (*n*)	10	13	0.318
Fontaine IV (*n*)	96	80	0.318
Baseline ankle brachial pressure index	0.43 ± 0.28	0.44 ± 0.21	0.831
Ulcer known since (d)	138 ± 147	187 ± 323	0.258
Diabetes mellitus (*n*)	64	61	0.562
Type 1 (*n*)	11	3	
Type 2 (*n*)	53	58	
Chronic kidney disease (*n*)	59	51	0.849
Arterial hypertension (*n*)	88	83	0.267
Coronary artery disease (*n*)	45	38	0.821
Chronic heart failure (*n*)	19	20	0.548
Stroke (*n*)	7	15	0.035
Cancer (*n*)	10	14	0.235
Chronic lung disease (*n*)	26	24	0.836
Atrial fibrillation (*n*)	28	25	0.973
Smoker (*n*)	17	22	0.067
Ex-Smoker (n)	81	60	
Oral anticoagulation (*n*)	31	29	0.396
Warfarin (*n*)	16	5	
Rivaroxaban (*n*)	6	7	
Apixaban (*n*)	9	15	
Metformin (*n*)	28	44	0.002
Insulin (*n*)	36	27	0.457
Aspirin (*n*)	48	32	0.119
Clopidogrel (*n*)	42	32	0.418
Rivaroxaban 2.5 mg BID (*n*)	4	4	
Statin (*n*)	54	71	<0.001
Ezetimibe (*n*)	0	0	
PCSK9 (*n*)	1	1	
Angiotensin-converting enzyme inhibitor/Angiotensin receptor blocker (*n*)	57	62	0.145
Beta blocker (*n*)	34	39	0.236
Diuretic (*n*)	2	0	0.325
Calcium channel blocker (*n*)	26	19	0.382

**Table 2:** Procedural characteristics pre- and during COVID. Values are mean and standard deviation (or percent of total if indicated); *p* values are from *t*-test or Mann–Whitney U test. Length of stay reported as median and interquartile range (IQR).

	Pre-COVID-19	During COVID-19	*p*
	07/2018–02/2020	03/2020–07/2021	
Iliac (*n*)	10	14	
Femoral (*n*)	67	55	
Popliteal (*n*)	34	42	
Crural (*n*)	63	47	
Number treated levels (*n* [1/2/3/4])	49/41/14/2	43/31/18/1	
Recanalisation (*n*)	87	79	
Total lesion length (mm)	141 ± 97	253 ± 158	<0.001
Stenting (*n* patients)	55	53	
Bare metal stents (*n* implanted)	49	45	
Drug eluting stents (*n* implanted)	36	39	
Total stent length (mm)	135 ± 95	87 ± 82	0.005
Drug coated balloons (*n* patients)	18	49	
Total length drug-coated balloon (mm)	157 ± 98	163 ± 123	0.815
Fluoroscopy time (s)	1294 ± 1010	1398 ± 756	0.450
Contrast volume (mL)	89 ± 40	97 ± 37	0.187
Technical success	92%	91%	0.593
Post procedure ankle brachial pressure index	0.81 ± 0.19	0.87 ± 0.20	0.045
Delta ankle brachial pressure index (post-baseline)	0.37 ± 0.31	0.42 ± 0.22	0.210
Increased ankle brachial pressure index (increased/unchanged/decreased)	91%/6%/3%	96%/3%/1%	0.183
Minor procedure related complication (*n*)	5	3	0.594
Minor	5%	3%	
Spurious aneurysm (*n*)	0	0	
AV fistula (*n*)	0	0	
Hematoma (*n*)	5	3	
Major/Life threatening (*n*)	0	0	
Length of stay (d, (IQR))	36 (46)	25 (36)	<0.001

**Table 3:** The 30-day outcomes of patients receiving angioplasties for CLTI pre- and during COVID-19. Values are the number of cases with respective endpoint (for all patients and stratified by day case/hospitalised; percentage next to event refers to cumulative survival at time of last event); *p* value is from the log-rank (Mantel–Cox) test.

	All	Pre-COVID-19	During COVID-19	*p* (Log Rank Mantel-Cox)
	Events	07/2018–02/2020	03/2020–07/2021	
**30-day wound healing**	11 (7%)	5 (6%)	6 (9%)	0.911
Day cases	11 (12%)	5 (12%)	6 (12%)	0.911
Hospitalised	0	0	0	
***p* (log rank Mantel-Cox)**	0.010	0.024	0.169	
**30-day mortality**	9 (4%)	4 (4%)	5 (6%)	0.558
Day cases	4 (4%)	1 (2%)	3 (1%)	0.451
Hospitalised	5 (7%)	3 (6%)	2 (10%)	0.538
***p* (log rank Mantel-Cox)**	0.230	0.333	0.294	
**30-day amputation**	2 (1%)	0	2 (3%)	0.212
Day cases	2 (2%)	0	2 (4%)	0.212
Hospitalised	0	0	0	
***p* (log rank Mantel-Cox)**	0.287		0.469	
**30-day target lesion revascularisation**	4 (2%)	2 (2%)	2 (2%)	0.734
Day cases	2 (2%)	1 (2%)	1 (1%)	0.866
Hospitalised	2 (3%)	1 (2%)	1 (5%)	0.461
***p* (log rank Mantel-Cox)**	0.568	0.866	0.488	

**Table 4:** One-year outcome of patients receiving angioplasties for CLTI pre and during COVID-19. Values are the number of cases with respective endpoint (for all patients and stratified by day case/hospitalised; percentage next to event refers to cumulative survival at time of last event); *p* value is from the log-rank (Mantel–Cox) test. (TLR = target lesion revascularisation).

	All		Pre-COVID-19		During COVID-19		*p* (Log Rank Mantel-Cox)
			07/2018–02/2020		03/2020–07/2021		
	Events (*n*)	Median (95%CI)	Events (*n*)	Median (95%CI)	Events (*n*)	Median (95%CI)	
**1-year wound healing**	98 (83%)	169 (132, 205)	52 (83%)	194 (141, 247)	46 (83%)	160 (114, 206)	0.746
Day cases	65 (83%)	168 (129, 207)	31 (85%)	168 (75, 261)	34 (81%)	169 (119, 219)	0.666
Hospitalised	33 (84%)	194 (139, 249)	21 (81%)	198 (123, 273)	12 (90%)	160 (31, 289)	0.234
***p* (log rank Mantel-Cox)**	0.609		0.248		0.493		
**1-year mortality**	47 (25%)	297 (280, 315)	27 (26%)	294 (271, 318)	20 (24%)	300 (274, 326)	0.381
Day cases	17 (14%)	330 (314, 347)	5 (10%)	343 (323, 362)	12 (18%)	319 (294, 344)	0.268
Hospitalised	30 (43%)	239 (205, 274)	22 (44%)	241 (202, 281)	8 (40%)	235 (166, 305)	0.913
***p* (log rank Mantel-Cox)**	<0.001		<0.001		0.012		
**1-year amputation**	12 (7%)	346 (335,357)	3 (3%)	357 (347, 366)	9 (12%)	332 (311, 353)	0.024
Day cases	8 (7%)	345 (331, 359)	1 (2%)	359 (347, 371)	7 (12%)	333 (309, 357)	0.048
Hospitalised	4 (8%)	346 (327, 364)	2 (7%)	352 (335, 369)	2 (13%)	327 (278, 376)	0.271
***p* (log rank Mantel-Cox)**	0.510		0.360		0.873		
**1-year TLR**	12 (7%)	345 (333, 356)	11 (12%)		1 (1%)		0.007
Day cases	9 (8%)	343 (328, 357)	8 (15%)		1 (2%)		0.012
Hospitalised	3 (5%)	349 (332, 367)	3 (6%)		0		0.309
***p* (log rank Mantel-Cox)**	0.236		0.272		0.617		

**Table 5:** Cox regression analysis to determine significant contributors of mortality (ABPI = ankle brachial pressure index).

	Hazard Ratio (95% CI)	*p*
**Age**	1.05 (1.01, 1.08)	0.014
**Male sex**	0.95 (0.48, 1.89)	0.894
**Hospitalised**	4.50 (1.94, 10.40)	<0.001
**ABPI baseline**	1.10 (0.32, 3.76)	0.884
**Time to procedure**	1.00 (0.97, 10.3)	0.879
**During COVID-19**	1.35 (0.70, 2.63)	0.369
**Diabetes mellitus**	1.14 (0.50, 2.59)	0.749
**Coronary artery disease**	1.99 (1.02, 3.91)	0.044
**Statin**	1.52 (0.73, 3.14)	0.259

## Data Availability

As this was part of an NHS service evaluation, the data are not publicly available.
